# Rapid prospective motion correction using free induction decay and stationary field probe navigators at 7T

**DOI:** 10.1002/mrm.30441

**Published:** 2025-01-23

**Authors:** Matthias Serger, Rüdiger Stirnberg, Philipp Ehses, Tony Stöcker

**Affiliations:** ^1^ MR Physics German Center for Neurodegenerative Diseases (DZNE) Bonn Germany; ^2^ Department of Physics & Astronomy University of Bonn Bonn Germany

**Keywords:** 3D‐EPI, FID navigators, NMR field probes, prospective motion correction

## Abstract

**Purpose:**

MR‐based FID navigators (FIDnavs) do not require gradient pulses and are attractive for prospective motion correction (PMC) due to short acquisition times and high sampling rates. However, accuracy and precision are limited and depend on a separate calibration measurement. Besides FIDnavs, stationary NMR field probes are also capable of measuring local, motion‐induced field changes. In this work, a linear model is calibrated between field probe data and motion parameters analog to FIDnav calibration and both tracking methods are compared and combined for PMC.

**Methods:**

FIDnavs and field probe navigators were implemented in a fast 3D‐EPI sequence and calibrated by a linear model to realignment motion parameters of the 3D‐EPI time series. A workflow was established to correct head motion prospectively by FIDnavs, field probe navigators or a combination of both. Large motions were instructed to test the accuracy and the impact on image quality in $1\;{\text{mm}}^{3}$ EPI data.

**Results:**

In a group of five subjects, FIDnavs demonstrated approximately doubled accuracy and precision in comparison with field probe navigators for large motions, especially nodding motions were tracked less accurately by field probes. A combination of both methods could not improve the accuracy consistently. Motion artifacts in high‐resolution data were reduced similarly by both PMC methods, although artifacts remained due to susceptibility‐induced B0 changes.

**Conclusion:**

Stationary field probe navigators can be calibrated equivalently as FIDnavs and enable rapid PMC of large and fast motions. Although they reveal decreased accuracy, their contrast‐independence facilitates the potential insertion into many sequences.

## 1 INTRODUCTION

Head motion during an MRI scan is a serious confound and can cause artifacts, preventing diagnosis and further image analysis. The level of degradation depends on multiple factors, including motion amplitude and timing as well as image resolution and k‐space sampling strategy.^1^ However, head motion is relatively simple to model by a rigid body with 6 degrees of freedom (DOF) and the effect on k‐space is also well‐known: head rotations cause rotations in k‐space and head translations cause phase shifts in k‐space. These changes can be corrected retrospectively,^2^ prospectively^3^ or by a combination of both.^4^ The prospective approach is more favorable in terms of k‐space consistency but it relies on a motion tracking system with high temporal resolution, low latency, and accurate and precise motion measurements. External systems (e.g., optical cameras with^5^, ^6^ and without optical markers^7^ or NMR markers^8^, ^9^, ^10^, ^11^) are capable of providing motion parameters at very high temporal resolution. However, for methods that use markers, a strong coupling to the skull needs to be ensured which might decrease patient comfort. Optical cameras without markers^7^ represent an improvement to that, but current designs rely on a clear line of sight to a part of the eye region, which is not available with most 7 T head coil designs.

On the other hand, motion estimates can also be determined by interleaved rapid MRI measurements using the scanner hardware itself (RF coils and imaging gradients). However, such MR image‐based navigators require sufficient dead time in the MR sequence. In this regard, the most demanding navigators acquire a whole 3D image (or a plane in 2D imaging) during a free gap in the sequence (e.g., vNavs^12^, ^13^). Then, the series of images is registered to provide motion estimates. Besides the low temporal resolution, they are computationally demanding for PMC, which can increase the latency. Alternatively, only the fat signal can be imaged, which permits higher acceleration factors (FatNavs^14^, ^15^). Typically, spatial resolution of MR image‐based navigators is relatively coarse, but the precision of motion estimates obtained by image registration is known to be much higher.^16^

As the effect of motion in k‐space is already known, the image reconstruction stage can be skipped to derive motion estimates directly from so‐called k‐space navigators, which can be acquired in less than 5 ms to give accurate motion estimates. Different methods are available to sample trajectories in k‐space using short imaging gradient sequences (e.g., orbital,^17^ cloverleaf,^18^ or spherical navigators^19^) that decode motion from signal changes in k‐space. Recently, a new model (servo navigators with 3D orbital trajectory^20^) was proposed that unites minimal calibration, short acquisition time (2.3 ms) and fast run‐time computation. For many applications, it seems to be a promising approach.

Without imaging gradients, it is possible to reduce the acquisition time even further and exploit the inherent spatial information of the multichannel head coil array. These free induction decay navigators (FIDnavs^21^, ^22^) can be sampled in less than 100 μs and inserted in almost every sequence without disturbing the magnetization. However, the relation between FIDnavs and motion is subject‐specific and demands a separate calibration scan^23^ or reference image measurement together with a simulation step.^22^ Furthermore, the FIDnav signal dependence on the imaging contrast may complicate application across different sequences.

In previous studies,^23^ FIDnavs were used to calibrate a motion model based on tracking data from external systems which necessitates the purchase and installation of these systems. Furthermore, it may introduce errors. Instead, in this work, low‐resolution 3D‐EPI images (equivalent to image‐space navigators^13^) are used to provide motion parameters for calibration.

Besides FIDnavs that measure intracranial field changes, external NMR field probes close to the imaging volume are sensitive to (extracranial) field changes induced by head motions. Probes attached directly to the head^10^, ^24^ proved to be a highly precise tracking method at the expense of decreased patient comfort. Stationary probes attached to the receive coil are also capable of measuring motion‐induced field changes in a gradient‐free window.^25^ Although the true relation between probe signal changes and motion is complex, it can be linearized for small motions, which is also valid for FIDnavs. Because these field probe navigators are similar to FIDnavs in that they measure subject‐specific, position‐dependent, unencoded MR signals that need to be calibrated by a linear model to motion, an equivalent modeling for motion tracking is tested in this work. Furthermore, both tracking methods are integrated into a PMC workflow and tested on a high‐resolution sequence.

## 2 METHODS

### 2.1 Data acquisition and processing

**FIGURE 1:**
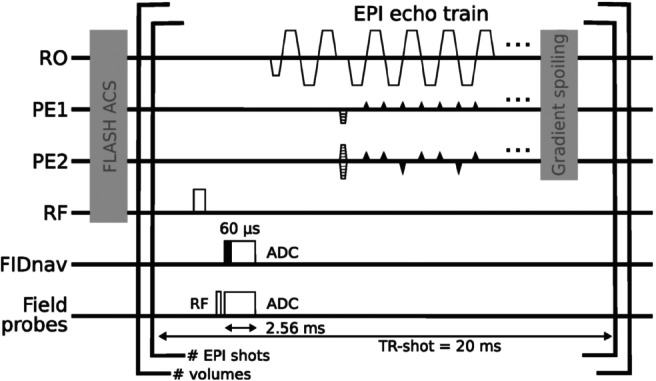
Modified 3D‐EPI sequence diagram. FIDnavs and field probe navigators are acquired every TR‐shot following the RF excitation pulse during a gradient‐free time window. Only the first 60 μs of the FIDnav readout were considered. Phase navigators per shot were employed to account for changes in the misalignment of even and odd echoes caused by large rotations of the readout gradient during PMC for substantial patient movements.

A FIDnav readout (2.56 ms, 512 samples, 5 μs dwell time) was inserted in a gradient‐free window after each RF excitation pulse of a multi‐shot 3D‐EPI sequence^26^ (Figure 1). To avoid filter transients, four samples from the start and end of the ADC were removed. The remaining 504 samples per FIDnav measurement can be averaged, although only the initial 4 samples (20 μs) proved to be most beneficial, as shown below. From the average FID signal (DC component of the corresponding spectrum), the real and imaginary parts are used independently as navigator values.^23^ Therefore, the 32 channels of the receive coil provided 64 values per FIDnav measurement. Independently excited FID signals of 16 stationary NMR field probes^27^ (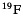, T1=80ms, T2=50ms, $\frac{\gamma_{F}}{2\pi }=40.05\;\text{MHz}⁄T$) were simultaneously acquired during the same gradient‐free interval. Local B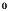 estimates were then obtained by a linear fit of the phase data of the fluorine samples, providing 16 field probe navigator samples.

Two protocols were set up (Table 1): one with low spatial and high temporal resolution primarily used to acquire fast image time series for calibration and one with high spatial resolution to test the impact of the correction methods on image quality. In addition to providing ground truth motion parameters for calibration, the time series protocol was also used to validate the accuracy of the correction methods. Note, however, that one image is obtained every 21 shots (0.42 s). To obtain ground truth motion for each of the 21 FIDnavs per volume, the image volumes were assigned to the 14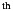 FIDnav per volume (k‐space center shot) and linearly interpolation was performed between volumes. The same shot repetition time (TR‐shot) and the same flip angle were chosen for both protocols. Otherwise, differing steady‐state signals (tissue $T_{1}$‐contrast) might affect the FIDnav performance.^22^ All scans use sagittal slice orientation and a single, non‐selective water excitation pulse of 1.02 ms duration.^28^

**TABLE 1:** $T_{1}$‐contrast‐matched 3D‐EPI protocols used for calibration and testing.

Sequence	Time series	High‐res.
Isotropic resolution (mm)	3	1
Base resolution	80	240
Slices	56	156
TR‐shot (ms)	20	20
Shots/volume	21	780
TA/volume (s)	0.42	15.6
FA (°)	5	5
TE (ms)	11.3	12.6
Readout bandw. (Hz/Pixel)	2314	1302
Partial Fourier	7/8 × 6/8	1 × 1
Parallel Imaging	3 × 2	3 × 2
Slice CAIPI shift	1	1
In‐plane segmentation factor	‐	10
EPI factor	24	8
Echo spacing (ms)	0.54	1.05
Phase enc. bandw. (Hz/Pixel)	69	119

The field probes were originally developed for concurrent field monitoring which benefits from longer relaxation times. The short TR (20 ms) of our setup would necessitate less frequent probe excitations (e.g., every 8 shots/160 ms) to avoid signal saturation. However, this would impair PMC performance by fewer updates and a slow probe‐excitation rate. Exciting the field probes every TR‐shot, on the other hand, leads to a decreased signal‐to‐noise ratio (SNR), which may reduce the measurement precision (high probe‐excitation rate). Potentially, also spurious spin echo and stimulated echoes can occur, which may decrease the measurement accuracy and precision. After an initial investigation of the trade‐off between PMC update rate and model accuracy/precision, we decided to acquire data for every TR‐shot. This approach was motivated by observing that averaging high probe‐excitation‐rate data yielded comparable accuracy and precision to using a slower excitation rate.

### 2.2 Linear model calibration

The relation between FIDnavs or field probe data and motion can be modeled linearly in case of small motion. Due to variations in head size and shape as well as in the shim fields, it is furthermore limited to subject‐ and scan‐specific models, which require an individual calibration.^23^, ^25^ Ground truth motion parameters (6 DOF) were here obtained by registering the 3D‐EPI image series of the calibration scan retrospectively using MCFLIRT^16^ (registration accuracy: RMS≲0.1mm), similar to the principles of vNavs and FatNavs. Then, the registration parameters were linearly interpolated to match the temporal resolution of the FIDnavs and field probe B‐field measurements (navigator data) and temporally shifted to the k‐space center shot which contributes to most of the image contrast. Thus, the ground truth motion time series samples increased by a factor of shots per volume (21), which was found to improve the model accuracy compared with considering only navigator data acquired at the k‐space center shots. After subtracting respective time series means from the input variables (navigator data: X) and output variables (interpolated motion parameters: Y), the linear least squares fit was conducted using Equation (1).

(1) $$\begin{array}{l} \hat{\beta }={\left(X^{T}X\right)}^{-1}X^{T}Y\; \end{array}$$

X either consisted of the 64 FIDnavs real and imaginary parts or the B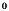 estimates of the 16 field probes. Additionally, a combined model of FIDnavs and field probes was tested, which then includes 80 input variables (64 FIDnavs, 16 B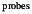).

The ability of the linear model to extrapolate motions, which are not included in the calibration set, is limited. For FIDnav‐based regression models, the generalization could be increased using a combination of two complementary motions (shaking (R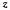) and nodding (R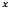)) for calibration.^23^ Due to the similarity of both methods and an initial investigation of this question, we assume this also holds true for field probe navigators.

We assess the model accuracy by two metrics: the root mean square deviation (RMS deviation^29^) and the mean absolute error (MAE) between ground truth motion and model prediction. Both time series start at zero. While the MAE can be used per motion parameter, the RMS deviation summarizes the difference between two ground truth and navigator‐derived transformations in a single metric (rotations are projected on a sphere with 80 mm radius). The RMS metric can be further used to quantify the magnitude of the ground truth motion using the unity transform (no motion) as a reference. Additionally, the standard deviation of the error (${\text{STD}}_{\text{err}}$) is used as a metric to quantify the precision.

Normalized root mean squared error (NRMSE) and structural similarity index (SSIM) were determined to quantitatively assess the image quality of the high‐resolution images. They were calculated after registration to a reference image acquired without PMC and with no intentional motion.

### 2.3 Prospective motion correction

#### 2.3.1 PMC workflow

**FIGURE 2:**
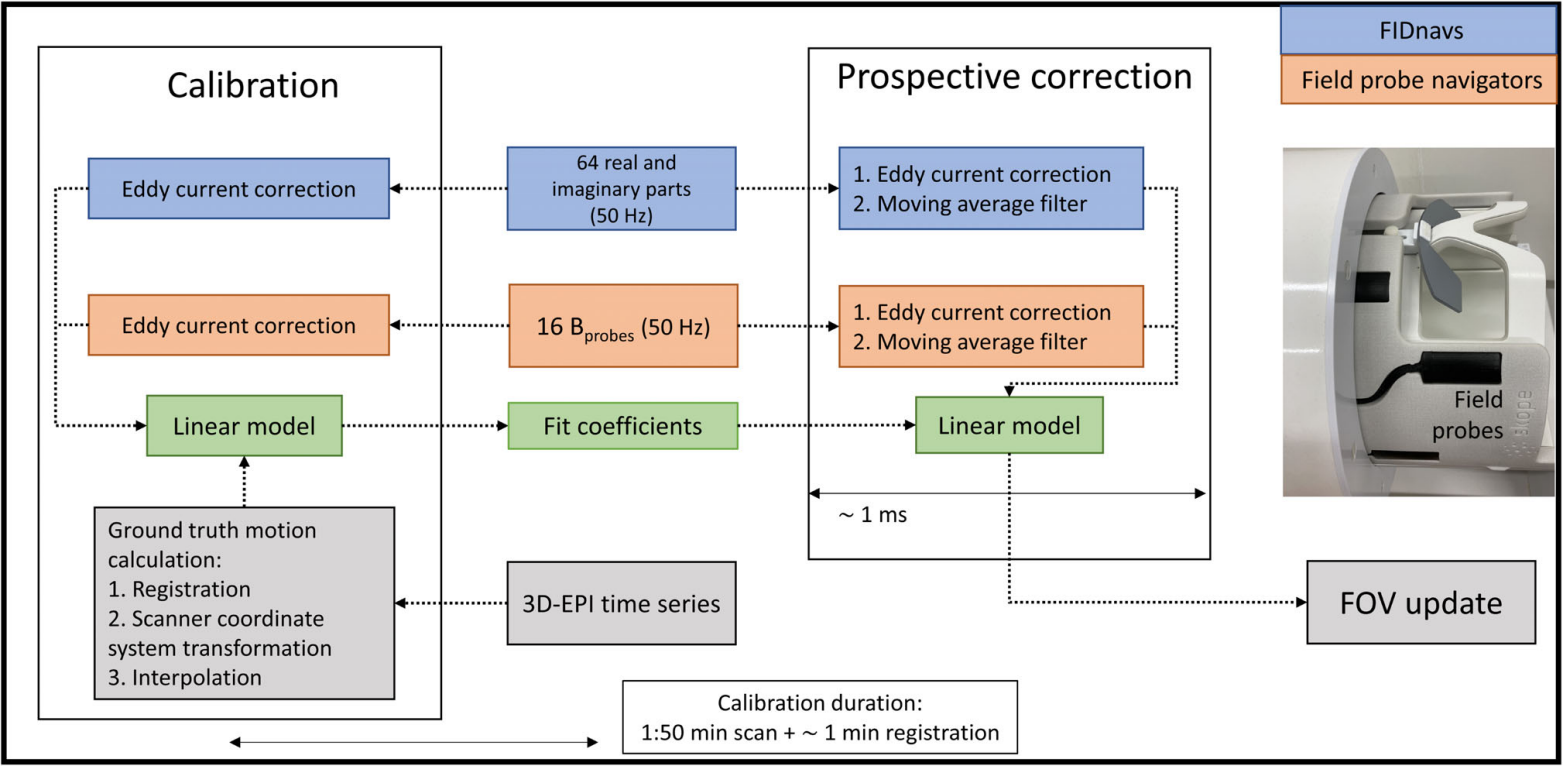
PMC workflow: FIDnavs and field probe navigator data were streamed to a server to calibrate a model online that is used in subsequent experiments for PMC. The moving average filter was only used in the 1 mm experiments to reduce eddy‐current induced variations.

FIDnavs and field probe data were streamed to a server which was used to calibrate the model online. The navigator data were received in two separate processes, allowing a separate and combined usage of FIDnavs and field probe data for PMC. For calibration, the simultaneously acquired 3D‐EPI images were transferred and registered online as well. The resulting motion parameters (stored as affine matrices using MCFLIRT's‐mats option) were then transformed from the image space to the scanner coordinate system. After the model was calibrated using navigator and scanner coordinate motion parameters, it was used in subsequent scans to predict head motions under the assumption that the shim fields of the calibration scan were retained. The server sends corresponding motion updates to the scanner for PMC (Figure 2).

We used a library for real‐time motion correction (libXPACE^5^) that provides a network interface to receive motion updates. It updates the slice position and orientation to rotate the gradient waveforms according to the head orientation and adjusts the RF and demodulation frequency to compensate for translations. For PMC, a small latency and high update rate are crucial to avoid artifacts in case of large and fast motions. The linear model is favorable in these aspects, because the calculation of motion parameter updates is negligibly short (within 1 ms in our setup) and updates could be provided before the next TR‐shot (in less than 20 ms).

#### 2.3.2 Eddy current correction

Due to the short timing between imaging (and spoiler) gradients and navigator data acquisitions, the latter are susceptible to measure eddy‐current induced fields that vary with the sequence's phase‐encoding pattern. Besides a systematic variation per volume that was observed for both sequences in Table 1, the segmented high‐resolution 3D‐EPI revealed a fast oscillation per partition (repeating with the segmentation factor, every 10 shots). This is caused by varying eddy currents due to echo time shifting^30^ affecting the subsequent navigator. To compensate for such motion‐independent variations that decrease the tracking precision, the shot‐ and channel‐specific navigator variations were estimated and averaged during the first 10‐s of each scan, when no motion was instructed, and removed afterwards in real time. During the 10‐s period, no PMC update was performed. In addition, a moving average filter (size = 10 shots) was utilized during the high‐resolution EPI acquisitions to suppress residual signal variations. Furthermore, the slow variation per volume of the high‐resolution sequence was determined in a phantom scan, smoothed by a moving average filter and removed in real‐time during the in vivo measurements.

#### 2.3.3 Retrospective ground truth motion estimation during PMC scans

A major problem in comparing PMC methods is the unavailability of uncorrected imaging data during the PMC scan. Therefore, separate experiments must be conducted where motion is executed with consistent magnitude and timing, either to induce or prevent comparable image artifacts. Furthermore, if an external motion tracking system is not available, the ground truth motion during the PMC scan is usually inaccessible and experiments may lack comparability. In case of our low‐resolution time series PMC scan, an estimation of the ground truth motion is nevertheless retained by adding residual motion retrospectively obtained from the image time series to the FOV updates applied during the PMC scan. To this end, the PMC images were registered and the corresponding motion (per image volume) linearly interpolated to the PMC update rate (per shot). The applied FOV updates were smoothed by a moving average filter (size = 21 shots/volume) to match the actual temporal resolution of both time series.

### 2.4 Experiments

Five healthy subjects were scanned using a MAGNETOM 7T Plus scanner (Siemens Healthineers, Erlangen, Germany) equipped with a 32‐channel Rx (1Tx) head coil (Nova Medical Inc, Wilmington, Delaware) after obtaining written informed consent in accordance with the local ethics regulations. The field probes (Skope, Zürich) were attached on a custom holder^31^ and placed between receive and transmit coil (Figure 2). To ensure consistent motion across all experiments, subjects were instructed to mimic head movements shown in a simultaneously presented animated head movie. Instructed motion patterns consisted of: shaking and nodding motions (24 s per direction, 5 s hold in extreme positions) and “drawing” horizontal and vertical figures‐of‐8 with the nose (24 s per figures‐of‐8).

The following experiments were performed. Unless stated otherwise, the fast time series protocol (Table 1) was used:

1. Calibration (1:50 min, 250 volumes):
  twice shaking and nodding
2. Retrospective motion tracking (3:40 min, 500 volumes):
  - involuntary small motions (“resting”)
  - voluntary large motions:
    twice shaking, nodding, horizontal and vertical figures‐of‐8
3. PMC (using FIDnav, field probe navigator, and combined models):
  - voluntary large motions (1:50 min, 250 volumes):
    shaking, nodding, horizontal, and vertical figures‐of‐8
  - High‐resolution imaging (1:10 min, 4 volumes at 1 mm isotropic resolution):
    resting, resting, shaking, and nodding

The high‐resolution experiment was repeated without PMC. Two different figures‐of‐8 motion patterns were included to test the generalization of the linear model (calibrated on shaking and nodding only) for a variety of motion states that were not included in the calibration data. Note that these patterns are particularly demanding for PMC, because they represent fast and continuous motion with large amplitudes on all axes simultaneously.

## 3 RESULTS

### 3.1 Tracking accuracy and precision

**FIGURE 3:**
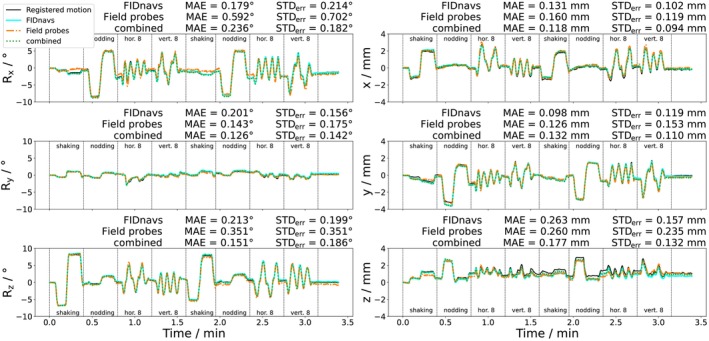
Registered ground truth and predicted motion time series of the second subject during the voluntary motion scan without PMC, performing shaking, nodding, and figures‐of‐8 motion trajectories twice. The comparison of three different tracking methods reveals that field probe navigators alone produce large errors for nodding ($R_{x}$) motions. The combination does not consistently lead to smaller errors.

In Figure 3, motion trajectories of representative voluntary motion (mean RMS ground truth motion: 3.86 mm) are compared across three methods: FIDnavs, field probe navigators and their combination. While the rotational errors (MAE) of the FIDnav‐based model show the same order of magnitude on all axes ($∼0.2^{\circ }$), the field probe model reveals a high error for nodding motions ($R_{x}$). In contrast, differences between all translation errors are small with slightly smaller values for FIDnav‐based models. Using a combination of both leads to an error reduction for some parameters ($R_{y}$, $R_{z}$, x, z) but not consistently for all parameters. The imprecision (STD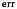) correlates with the error. Motions predicted by field probes systematically show a lower precision. Interestingly, the predictions of the z‐translation illustrate the sensitivity of MAE towards slow (unpredicted) drifts, while the STD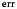 is more robust against slowly diverging ground truth and prediction.

**FIGURE 4:**
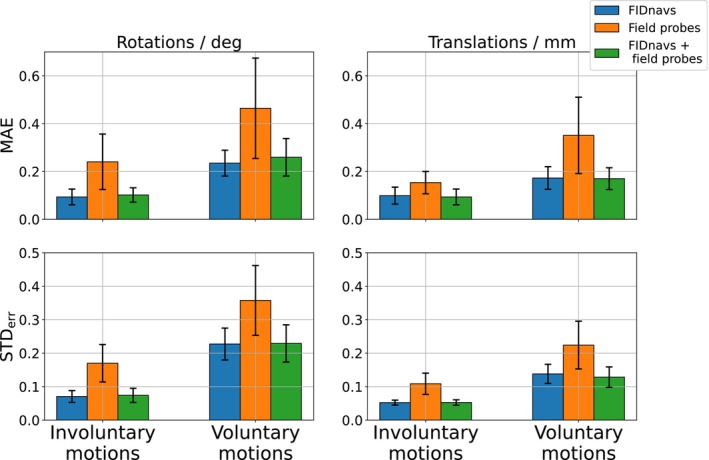
Group MAE and STD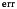 of all involuntary small and voluntary large motion scans. For both motion paradigms, linear models calibrated on FIDnavs show smaller prediction errors and higher precision in all metrics than models calibrated on field probe navigators alone.

In Figure 4, the group accuracy and precision of the methods are displayed for all retrospective motion tracking scans (without PMC). For involuntary small motions, FIDnav‐based regression models show decreased errors and higher precision across rotations (${\left(0.09\pm 0.07\right)}^{\circ }$) and translations ((0.10±0.05)mm) to field probe navigators (${\left(0.24\pm 0.17\right)}^{\circ }$ and (0.15±0.11)mm). The combination leads to minor improvements for translations ((0.09±0.05)mm), but rotations are not predicted more accurately by including field probe measurements (${\left(0.10\pm 0.07\right)}^{\circ }$). The increased magnitude of the errors in the voluntary motion experiment indicates that the error is scale‐dependent.

### 3.2 Prospective motion correction

#### 3.2.1 Residual motion analysis

**FIGURE 5:**
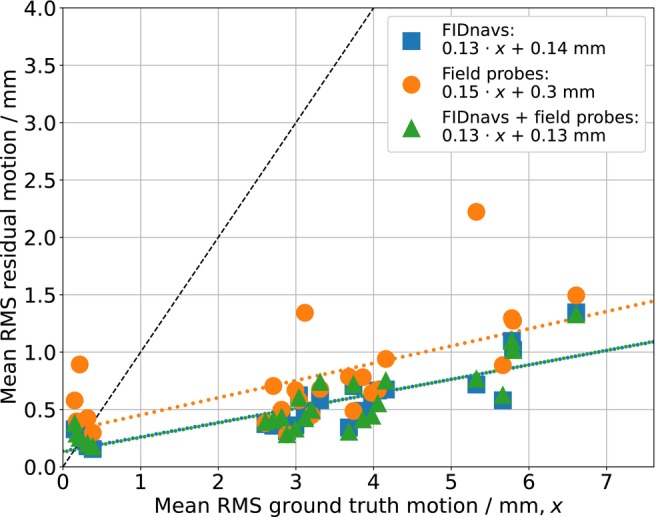
Mean RMS residual motion vs. mean RMS ground truth motion of all time series scans, including experiments without PMC. Errors increase with larger ground truth motion. Field probe predictions show larger errors in most cases. Extreme outliers can be seen for some of the field probe predictions. Values above the identity line (black) indicate that some of the scans with small, involuntary motion would not benefit from PMC.

Using the retrospectively estimated ground truth motion of the measurements with PMC, the accuracy of each method can be compared for all time series scans (Figure 5). Although the residual motion is small, it is not reduced to the levels observed in involuntary motion scans for most subjects. For more detailed information about the magnitude of registered motions of all time series scans, please refer to Supporting Information Figure S1. In Figure 5, a scale‐dependence of the error (mean RMS residual motion) can be observed. The error increases approximately linearly with the mean RMS ground truth motion (approximate slopes for FIDnavs/field probes/combined: 0.13/ 0.15/ 0.13). Values below the identity line (black) indicate a potential imaging improvement with PMC. While the errors predicted by FIDnavs and combined models show high similarity, the errors by field probe models reveal a few large outliers. Nevertheless, the errors would still be smaller in case of large motions than without PMC. On the other hand, small motion predictions (<0.5mm mean RMS) sometimes lead to relatively increased errors, particularly when using the field probe model. Residual errors in the absence of actual motion (intercepts) are extrapolated as: 0.14/ 0.30/ 0.13 mm.

**FIGURE 6:**
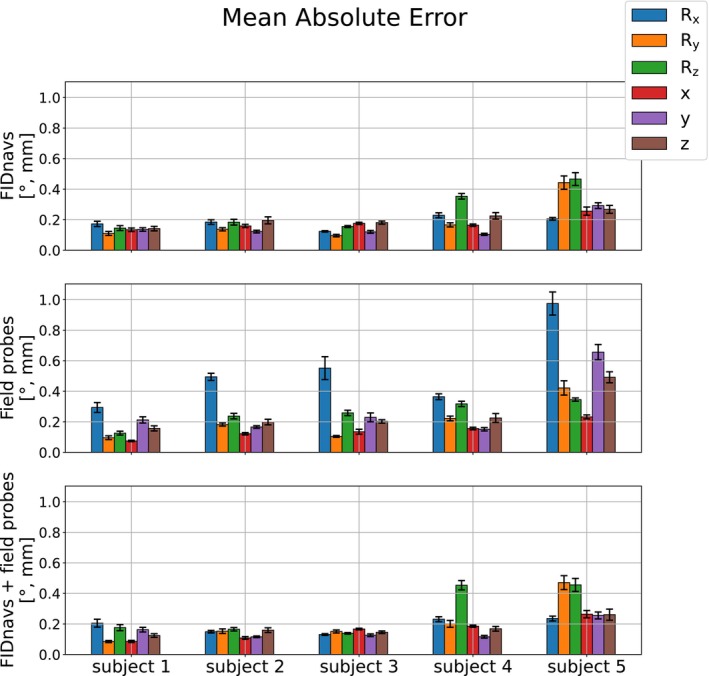
Averaged MAE of all low‐resolution scans, specified for each motion parameter. Field probe models yield large errors for nodding motions.

In Figure 6, the method‐specific bias for each motion parameter is displayed separately. The analysis reveals that the large rotational errors of field probe navigators are mainly observed for nodding motions ($R_{x}$). Apart from that, the other motion parameters show no substantial difference in the MAE across all subjects.

#### 3.2.2 PMC efficacy for high‐resolution data

**FIGURE 7:**
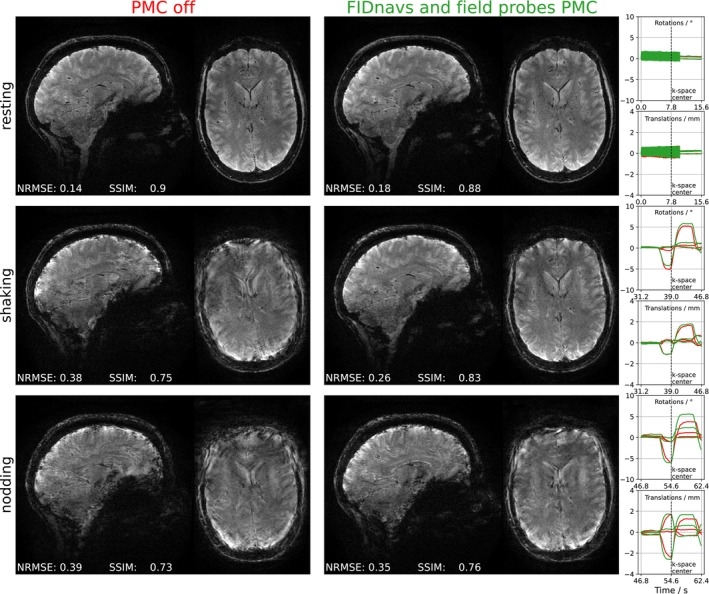
Sagittal and axial slices of uncorrected and prospectively corrected images (combined method) for three different motion paradigms: resting, shaking, and nodding. A reduction of artifacts can be observed with PMC, which is more pronounced for head shaking. Motion trajectories indicate similar timing and magnitude of motion that was instructed during k‐space center acquisition (vertical dotted line). The enhanced precision resulting from the eddy current correction is illustrated in the motion trace without instructed motion.

In Figure 7, the impact of PMC (combined model) on image quality is illustrated for two motion paradigms (shaking and nodding) in a single‐subject experiment. The predicted motion trajectories during the scans without and with PMC are also depicted to demonstrate comparable timing and magnitude of the performed motions. While the image quality without motion is not visibly reduced when PMC is turned on, artifacts in scans with large motion are noticeably reduced. Without PMC, strong blurring artifacts are visible for both shaking and nodding motions. Although the image quality is improved with PMC in both cases, some artifacts remain. In case of the shaking motion, ringing artifacts can be observed in the upper left region of the axial slice. Clearly more residual artifacts are visible in the corrected images acquired during nodding motion. Severe remaining blurring artifacts are visible in the frontal lobe and cerebellum. Compared with the other cases, almost as large NRMSE and as small SSIM values as without PMC support this observation.

SWI images and minimum intensity projections (mIP, 10 mm slab) were additionally calculated^32^ and are depicted in Figure S2. Although severe artifacts remain in the corrected images, the improvement in image quality allows for the identification of small blood vessels.

**FIGURE 8:**
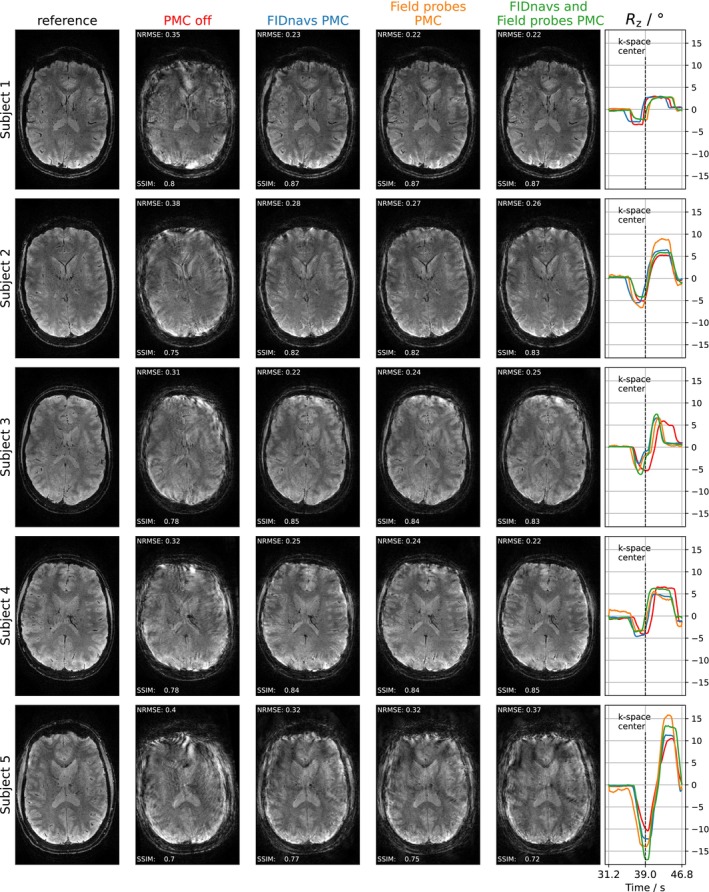
High‐resolution images acquired during the shaking motion paradigm of all subjects without and with PMC (FIDnavs, field probe navigators, and combined model). The reference image was obtained without motion and without PMC. For subjects 1–4, the image quality is similarly improved by all PMC methods compared with PMC off. According to the plotted FOV updates (or predicted motion for PMC off), Subject 5 performed a significantly larger and faster $R_{z}$ rotation compared with the other subjects. Here, the corrected images show severe remaining artifacts. The motion trajectory during PMC off was predicted by a combined model.

Figure 8 compares high‐resolution images acquired under the shaking motion paradigm across subjects and PMC methods. While the image quality is similarly improved by all PMC methods for motions $≲8^{\circ }$, the overall image quality is low for significantly larger rotations (see subject 5). Furthermore, the rotations performed by subject 5 in all PMC experiments are larger in amplitude and speed than during the experiment without any correction, which complicates a comparison. This analysis was repeated for the nodding experiments in Figure S3. For nodding motion, the improvements in image quality are less pronounced, supporting the results of Figure 7.

Extending the quantitative image quality analysis towards all high‐resolution scans (Figure S4), the errors (NRMSE) of prospectively corrected shaking motions are approximately 25−30% smaller on average (∼0.26) than for nodding motions (∼0.36). While all PMC methods reduce artifacts similarly for shaking motions, the field probe model results in highest errors and lowest similarity for nodding motions (only slightly exceeded without any correction), supporting the results of Figure 6.

## 4 DISCUSSION

### 4.1 Potential and challenges of stationary field probe navigators

Stationary field probe navigators can be used like FIDnavs to calibrate and predict subject‐specific head motion. However, decreased accuracy and precision of field probe navigators make this method appear inferior to FIDnavs under the experimental conditions examined here. Only minor improvements in FIDnav translation prediction could be achieved by a combined FIDnav and field probe navigator model. Note, however, that the probes were not optimally used to achieve the highest precision but to provide the highest update rate for PMC. This topic was investigated in a separate experiment, in which voluntary motions were instructed, while the probes were only excited every 160 ms (Figure S5). The results demonstrate that the more rapid excitation every 20 ms does not impact the rotational accuracy and precision. However, the error of translation prediction is increased by ∼30% and the precision is reduced by ∼40%. Nevertheless, for the present comparison, we chose rapid excitation because of its advantages for PMC with a high update rate. While dedicated motion correction field probes^24^ with very short T1 and T2 could be more beneficial for rapid PMC, the field probes used here allow for high precision and accuracy at lower update rates.

Another explanation for the performance difference between FIDnavs and field probes may be that the FIDnav real and imaginary signal components of 32 receive channels were utilized, while only the phase information of 16 field probes was used. Furthermore, the field probe positions could have a major impact on the method's performance. For static B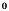 field measurements and field dynamics due to gradient switching, the 16 probes have been distributed as best as possible given the receive array geometry.^31^ With head motion, however, the specific probe positions relative to susceptibility‐induced field changes may also be important. For instance, head rotations orthogonal to the main magnetic field (e.g. nodding, $R_{x}$) cause particularly large changes that might become nonlinear at certain probe positions. As such, the outliers of the field probe models in Figure 5 originate from large errors of nodding motion prediction. Nevertheless, no substantial differences to FIDnav predictions were observed for rotations around other axes ($R_{y}$ and $R_{z}$).

If the observed weaknesses (nodding motions, low precision due to long T1 and T2) are sufficiently reduced, field probe navigators may be a promising motion correction technique that can be easily integrated into many sequences. In contrast to FIDnavs, they could also be utilized to detect out‐of‐plane motions in 2D sequences, and because they do not rely on the image contrast, the transfer to other sequences would be straightforward.

### 4.2 Minimal FIDnav acquisition

In all experiments, FIDnavs were sampled together with the field probe data for 2.56 ms. However, only the first 60 μs of the FIDnav (of which the initial and final 20 μs were additionally removed to avoid filter transients) were considered for the results presented here. In an initial investigation, we found that averaged short FIDnavs lead to smaller errors than long FIDnavs, especially for rotations. A retrospective analysis of all time series experiments with large motions for varying readout durations up to 2.56 ms underlines this observation (Figure S6). Rotation predictions improve by ∼30% and translation predictions improve by ∼10% when using 60 μs compared with 2.56 ms total ADC duration. As discussed before, rotations orthogonal to B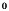 induce large magnetic susceptibility changes and a longer FIDnav readout (which enables the detection of lower frequencies) can be more sensitive toward these fields. Note that the change in the susceptibility field is a secondary effect of motion and it modulates the FIDnav signal without being considered in the motion model. Thus, motions could be estimated inaccurately when large susceptibility field changes are present. This is because, in contrast to field probes, each receive channel detects an FID signal from an extended head region. Strong B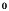 variations in such an extended field of reception lead to a complex FID time signal. Our findings suggest that particularly short ADC durations, which correspond to only 4 ADC samples over 20 μs in our experimental setup, make the FIDnav most robust. Note that such gradient‐free readouts are a large potential benefit of FIDnavs.

### 4.3 Validity of the linear model

The observed scale‐dependent errors in both methods indicate that the linearity of the model was violated by large motions. However, the linear increase is small compared with the increase in motion magnitude.

Due to these imperfections of the linear model, we investigated regularized linear models which are theoretically able to account for model bias to improve the generalization. Here, we only observed an improvement for very large motion amplitudes ($\geq 10^{\circ }$) or if long FIDnav readouts (2.56 ms) were used. For smaller motion amplitudes and short readouts, regularization leads to higher errors.

Another potentially interesting type of models that could provide fast run‐time computation and complex modeling are neural networks. However, they require a large train set for generalization and the training phase is computationally intensive, which is challenging in most scan scenarios. Although a generalized model for multiple subjects could solve this issue, inter‐subject and inter‐scan covariates need to be determined. Potential factors could be the head geometry and shim fields.^23^ Comparing the field changes for shaking motions inter‐subject‐wise (Figure S7), reveals that the slope between signal changes in some receive coils (field probes) and motion is linear but differs between subjects. The reason for this systematic difference could not be found and requires further investigation which exceeds the scope of this work. Even if the relation would be determined, the FIDnav dependence on the MR contrast would complicate a transfer to other protocols and sequences. For field probe navigators, a generalized model would not suffer from this MR contrast restriction.

### 4.4 Artifact reduction in high‐resolution 3D‐EPI images

Although the presented PMC methods were able to correct large and fast motions with moderate accuracy and precision, severe residual artifacts can be observed in the corrected 1 mm 3D‐EPI images in case of a nodding motion when compared with a shaking motion. This is despite the observation that FIDnavs (and combined) models provide increased accuracy for nodding motions compared with field probe‐only models. One possible explanation is that such susceptibility‐induced field changes persist, even though the FOV has been successfully updated. To explore this phenomenon in more detail, an additional experiment was conducted, acquiring a double‐echo 3D‐EPI time series with alternating echo times between image volumes (${\text{TE}}_{1}=10.8\;\text{ms}$, ${\text{TE}}_{2}=11.3\;\text{ms}$), similar to the calibration experiment. From this data, field maps were calculated dynamically and compared for large positions with PMC off and on (Figure S8). It can be observed that large field changes occur in the frontal lobe and the cerebellum for nodding motions even when PMC is on. These are also regions that showed the most severe blurring in the corrected images. This supports the conclusion that the reduced image quality compared with shaking motions mainly stems from susceptibility‐induced field changes. Fortunately, the field changes appear to be linear along the AP direction and could thus potentially be corrected by a first‐order shimming method. FIDnav‐based real‐time shimming has been demonstrated previously.^33^ However, simultaneous prospective correction of motion and B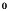 changes using FIDnavs or field probe navigators is particularly challenging because both models rely on the relationship between signal (phase) changes and motions. The calibrated models are potentially being invalidated by dynamic shimming. The impact of prospective shimming on the validity of the motion model exceeds the scope of this work and needs to be evaluated in further experiments.

Considering the corrected images that were acquired under shaking head motion, the improvement of the image error and similarity metric is approximately equal for all correction methods, however without achieving the quality of images without instructed motion. In addition to residual prediction errors on the order of the 1 mm voxel length for very large motion amplitudes (cf. Figure 6), this could be because the performed rotational motions were relatively fast ($∼5^{\circ }/s$ in Figure 7). Such high motion amplitudes and speeds are generally not observed in large population studies.^34^ Nevertheless, if one assumes an optimal latency of a single TR‐shot (20 ms), this speed leads to an additional head rotation of up to $∼0.1^{\circ }$ between navigator acquisition and subsequent FOV update. In case of the high‐resolution scan, the latency is even prolonged by the use of a moving average filter (size = 10 shots). This would potentially lead to a residual head rotation of up to $∼0.5^{\circ }$ between navigator acquisition and FOV update.

### 4.5 Limitations

The residual artifacts discussed in the previous section demonstrate the limitations of the proposed methods for particularly fast and large motions, which are rarely encountered in clinical practice. In this work, the experimental design was chosen to test the limits for PMC and amplify differences between the methods, which became obvious in tracking large nodding motions, for example. Nevertheless, the estimated accuracy of both methods ($∼0.1-0.2^{\circ }/\text{mm}$) for involuntary small motions readily permits the correction of images acquired with resolutions up to 1 mm. This represents typical clinical resolutions. Research protocols, on the other hand, often acquire higher‐resolution images, especially at 7T. For ∼0.5mm voxel sizes, the achieved accuracy may not be sufficient.

We presented an EPI‐based calibration that neither requires external motion tracking hardware to provide ground truth motion nor subject‐specific, channel‐wise coil sensitivity profiles to generate virtual calibration data.^22^ Nevertheless, the length of the performed calibration scan of about two minutes represents another practical limitation. Furthermore, the choreographed calibration motions cannot be conducted in studies with uncooperative subjects. However, it should be noted that the calibration can alternatively be performed using navigator data and ground truth motion obtained from conventional sequences with short volume TR (e.g., functional MRI) that are routinely acquired in various studies.^35^ Although the tracking accuracy is significantly reduced (Figure S9), large (intra‐volume) motion events may still be detected (e.g., using framewise displacement^36^) and potentially used for triggering reacquisition of corrupted data.

## 5 CONCLUSION

In this work, we present a novel method for the calibration of stationary field probe measurements to head motion and compare its performance with FIDnavs‐based motion tracking. Both tracking methods as well as a combined model were integrated into a PMC workflow at 7 Tesla. Tracking accuracies down to approximately $0.1-0.2^{\circ }/\text{mm}$ were achieved. PMC results indicate a substantial reduction of residual motion in registered EPI time series. Certain motions that cause large susceptibility‐induced field changes, such as nodding, are challenging for either model, which may be improved through simultaneous B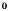 shimming. Although field probe navigators alone showed decreased accuracy, using dedicated field probes with shorter relaxation times and optimizing the spatial probe distribution may improve this method in the future.

## CONFLICT OF INTEREST STATEMENT

The authors declare no potential conflict of interests.

## FUNDING INFORMATION

This work received financial support from the European Union Horizon 2020 Research and Innovation program under grant agreement 885876 (AROMA).

## Supporting information




**Data S1.** Supporting Information.
