# Supplementary material for: Rapid prospective motion correction using free induction decay and stationary field probe navigators at 7T
Source: Magn Reson Med. 2025 Jan 23;94(1):105–18. doi: 10.1002/mrm.30441 (PMC12021338; doi:10.1002/mrm.30441)

Supporting Figure S1: Mean RMS ground truth motion of all time series scans. The ground truth motions during PMC were retrospectively estimated by adding the FOV updates to the residual, registered motion (light colors). The differences illustrate the effectiveness of the PMC methods. The retrospectively estimated motion parameters of PMC scans demonstrate that the magnitude of conducted motion paradigms was similar within subjects but varied between them. Although the residual motion (registered PMC images) is small, it is not reduced to the levels observed in involuntary motion scans for most subjects, except for subjects 1 and 2, where the use of FIDnavs alone or the combined model achieved better results.


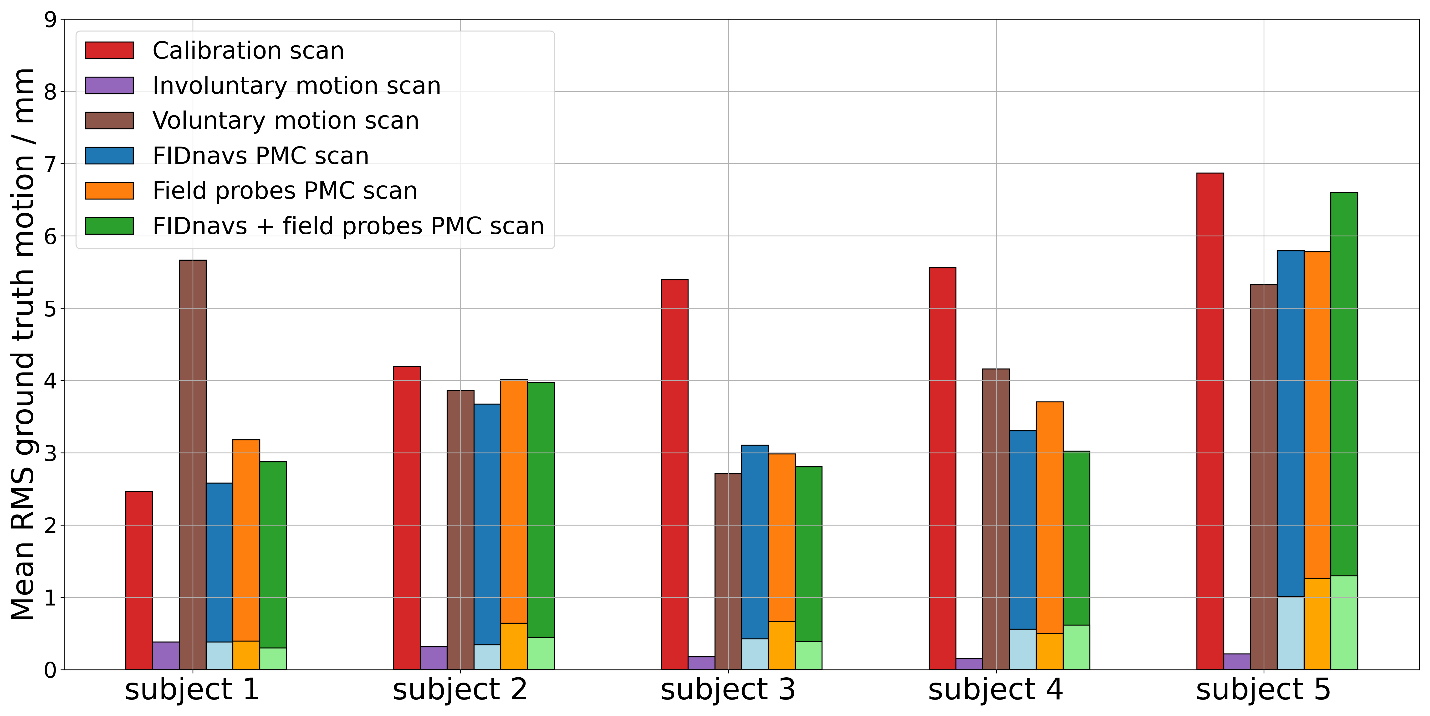


Supporting Figure S2: Susceptibility-weighted images (SWI) and minimum intensity projections (mIP, 10 mm slab) for different motion patterns during T 2 ∗ -weighted multi-shot 3D-EPI acquisition without and with PMC (magnitude images shown in Fig. 8). Blurring artifacts in the SWIs are significantly reduced with PMC and small blood vessels remain visible. For large nodding motion, dropouts are recognizable in the frontal lobe.


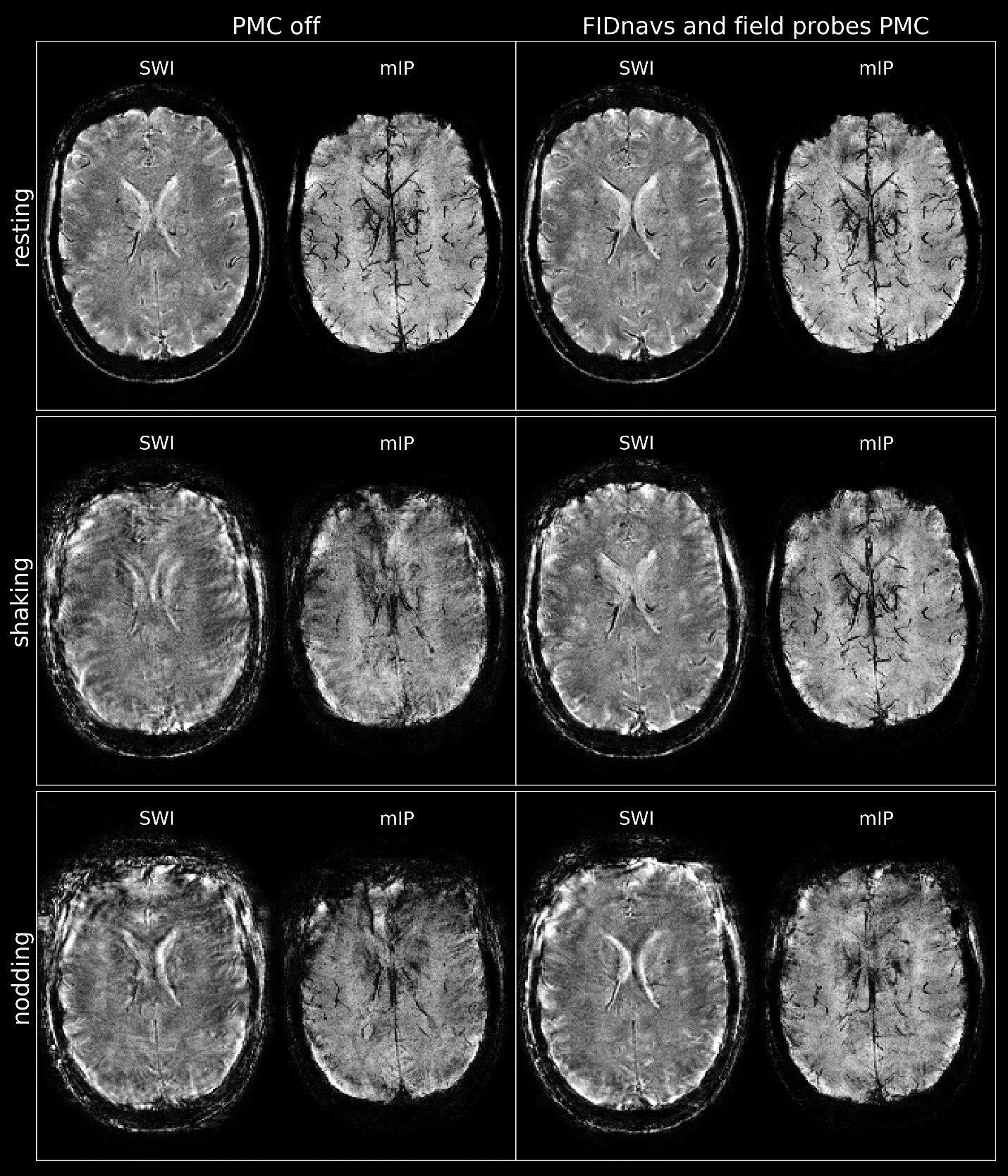


Supporting Figure S3: High-resolution images acquired during the nodding motion paradigm of all subjects without and with PMC (FIDnavs, field probe navigators and combined model). PMC only leads to minor improvements in image quality across all subjects. Subject 5 illustrates limitations when validating PMC methods: the performed nodding motions vary significantly in amplitude between corrected and uncorrected scans, which complicates a comparison. The motion trajectory during PMC off was predicted by a combined model.


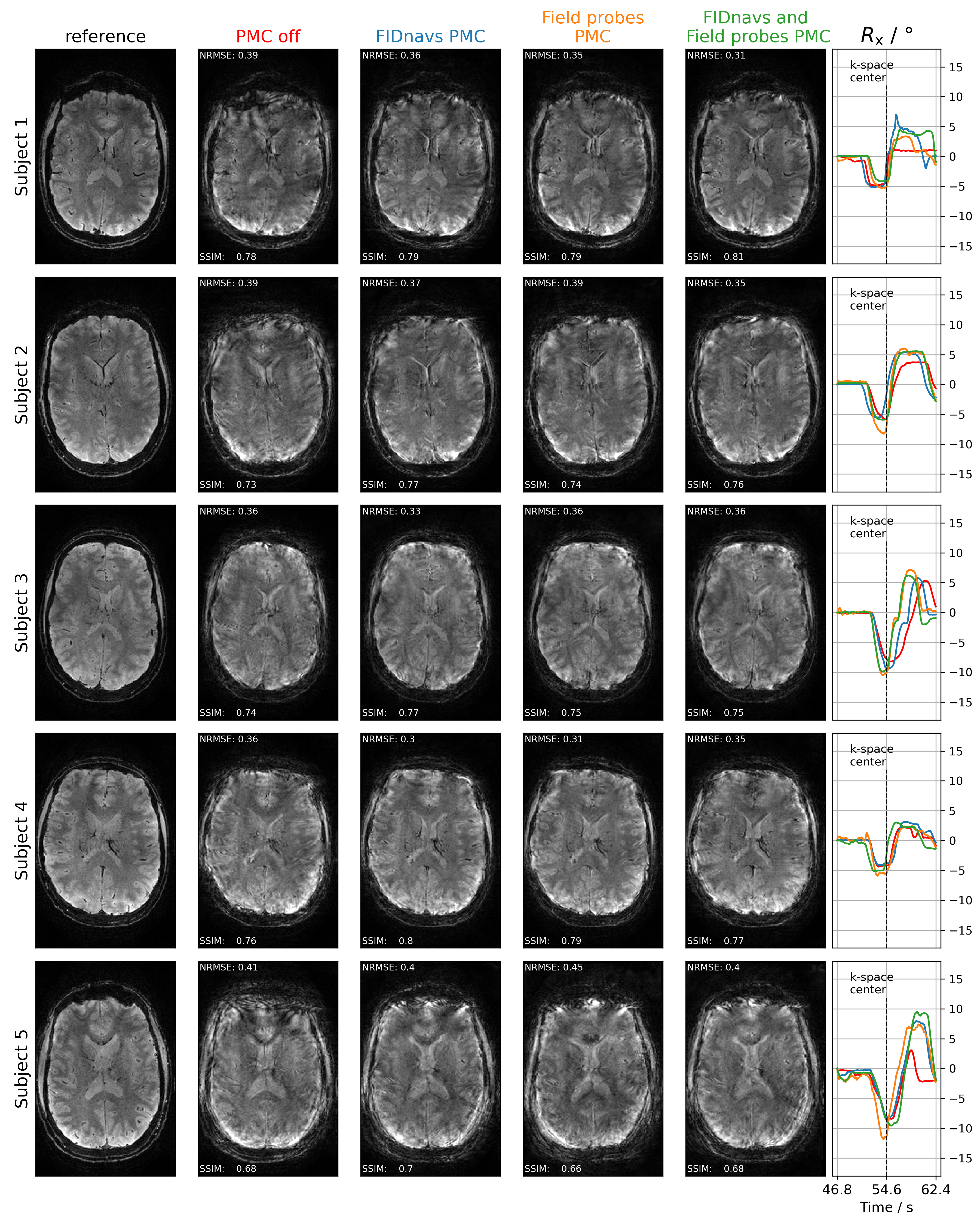


Supporting Figure S4: Group analysis of image quality metrics (NRMSE, SSIM) of all high-resolution scans without and with different PMC methods. With PMC on, images acquired during head shaking show smaller errors and higher similarity than those acquired during nodding motions. Although artifacts induced by shaking motions are reduced substantially through PMC with any model, the quality of PMC images without instructed motion is not achieved (average NRMSE ∼ 17 %). Apart from that, the best quality metrics of uncorrected images without instructed motion (second “resting” scan compared to the first “resting” scan) indicate inter-volume variations that cannot be further reduced by PMC (NRMSE ≳15 %, SSIM ≲ 0.9).


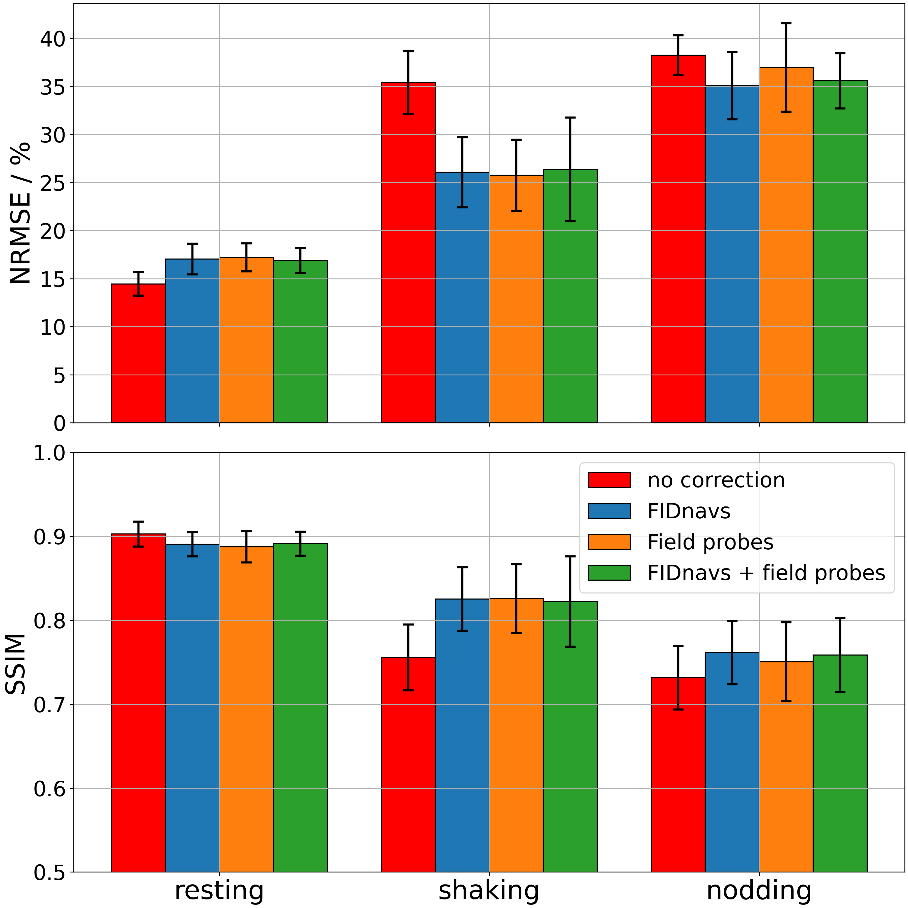


Supporting Figure S5: Comparison of field probe model predictions with rapidly excited probes (interleave repetition time, iTR = 20 ms) vs. a less frequent excitation (iTR = 160 ms) in a single subject experiment. Two separate models were trained for each iTR. The rapid measurements were filtered (moving average filter, window size 8 for the same effective temporal resolution) to demonstrate that the same rotational precision and accuracy can be achieved compared to less frequently excited probes. For translations, the accuracy is reduced by 30 % and the precision by 40 %.


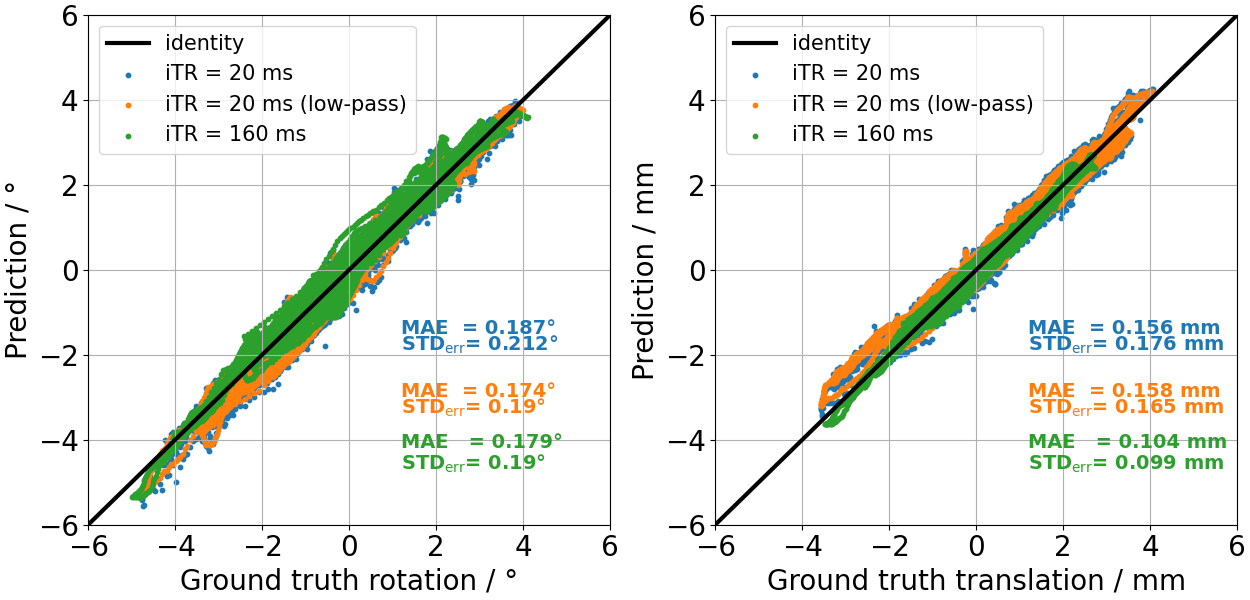


Supporting Figure S6: Impact of the FIDnav ADC duration on the model accuracy for all low-resolution scans with instructed large motions. While rotations improve by 30 % for optimal 60 µ s instead of 2.56 ms ADC duration, translations show smaller improvements of 10 %. Although the field probe errors are higher for translations, the combined model still benefits from the field probe information and a higher accuracy is achieved.


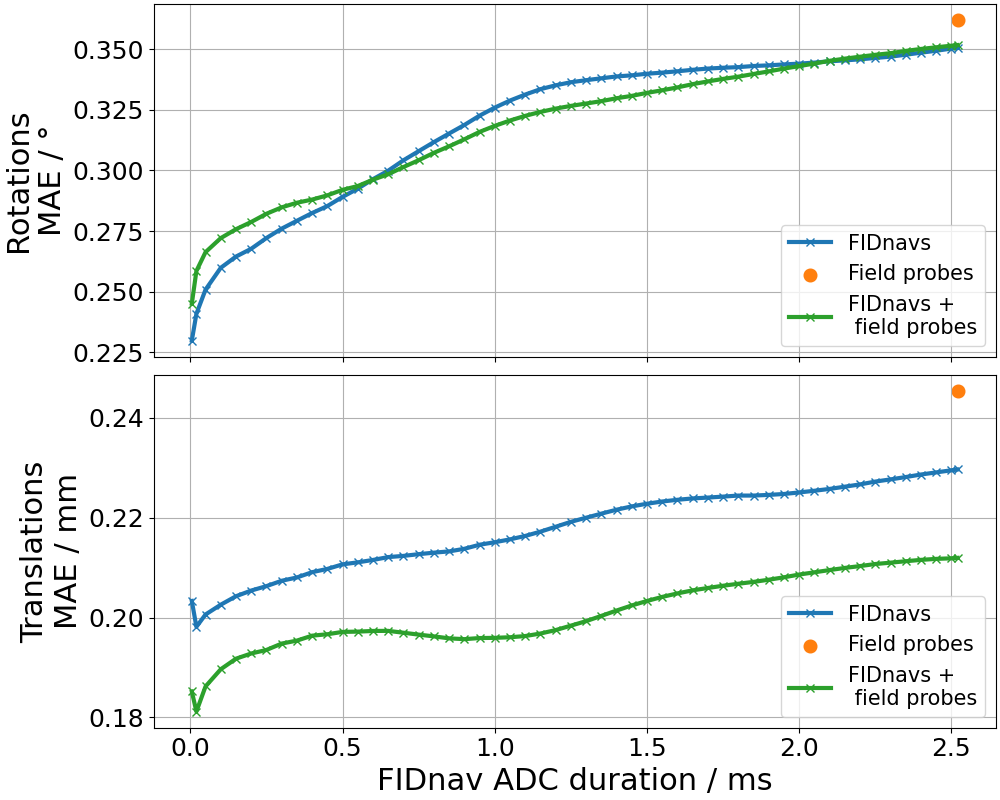


Supporting Figure S7: Relationship between signal changes and shaking head motions for a selected probe and coil. The observed linearity is subject-dependent. In some coils/field probes, the linearity was violated for large motions.


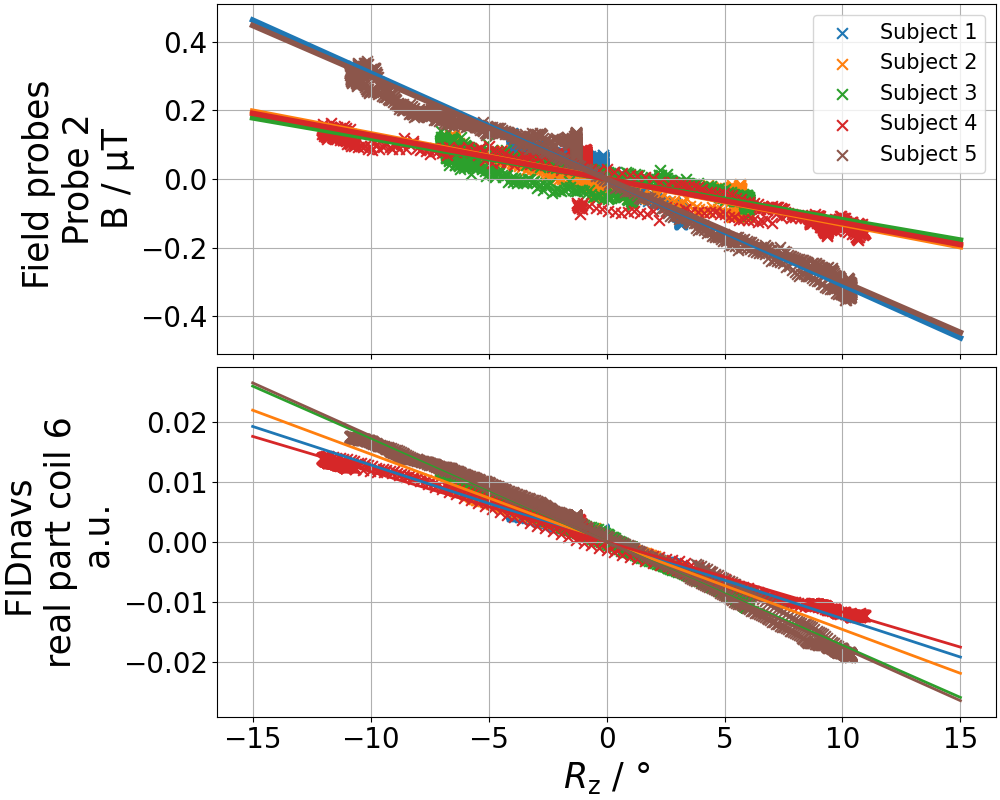


Supporting Figure S8: B0 field difference maps acquired for different example head poses (ground truth parameters indicated at the bottom of each subplot) during no motion, shaking and nodding motion patterns. A double-echo 3D-EPI (TE_1_=10.8 ms, TE_2_=11.3 ms) was utilized to measure dynamic field maps. All field maps were coregistered and subtracted from a field map without instructed motion to remove the common base line field. While shaking motion (around B0 axis) induces negligible field changes, nodding motion induces large field changes, especially in the frontal lobe and the cerebellum, regardless of PMC.


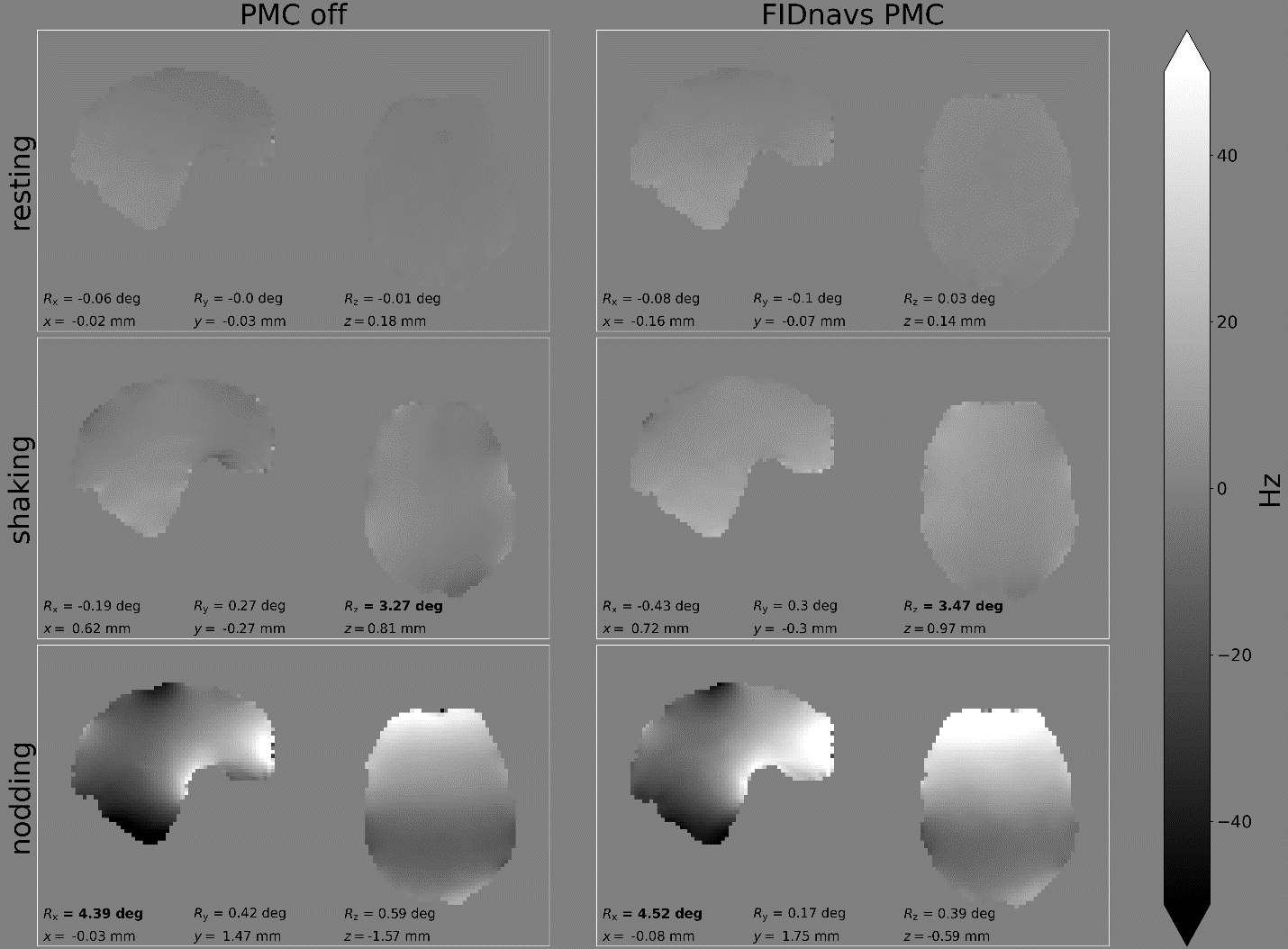


Supporting Figure S9: Predicted motion parameters of FIDnavs/field probe navigators calibrated on involuntary small motions vs. a calibration on large voluntary motions (test set: voluntary motions Subject 2). Large motions are underestimated by both models but the FIDnav-based model reveals smaller deviations. To obtain the same number of calibration samples, the data set with involuntary small motions (Subject 2) was divided in half.


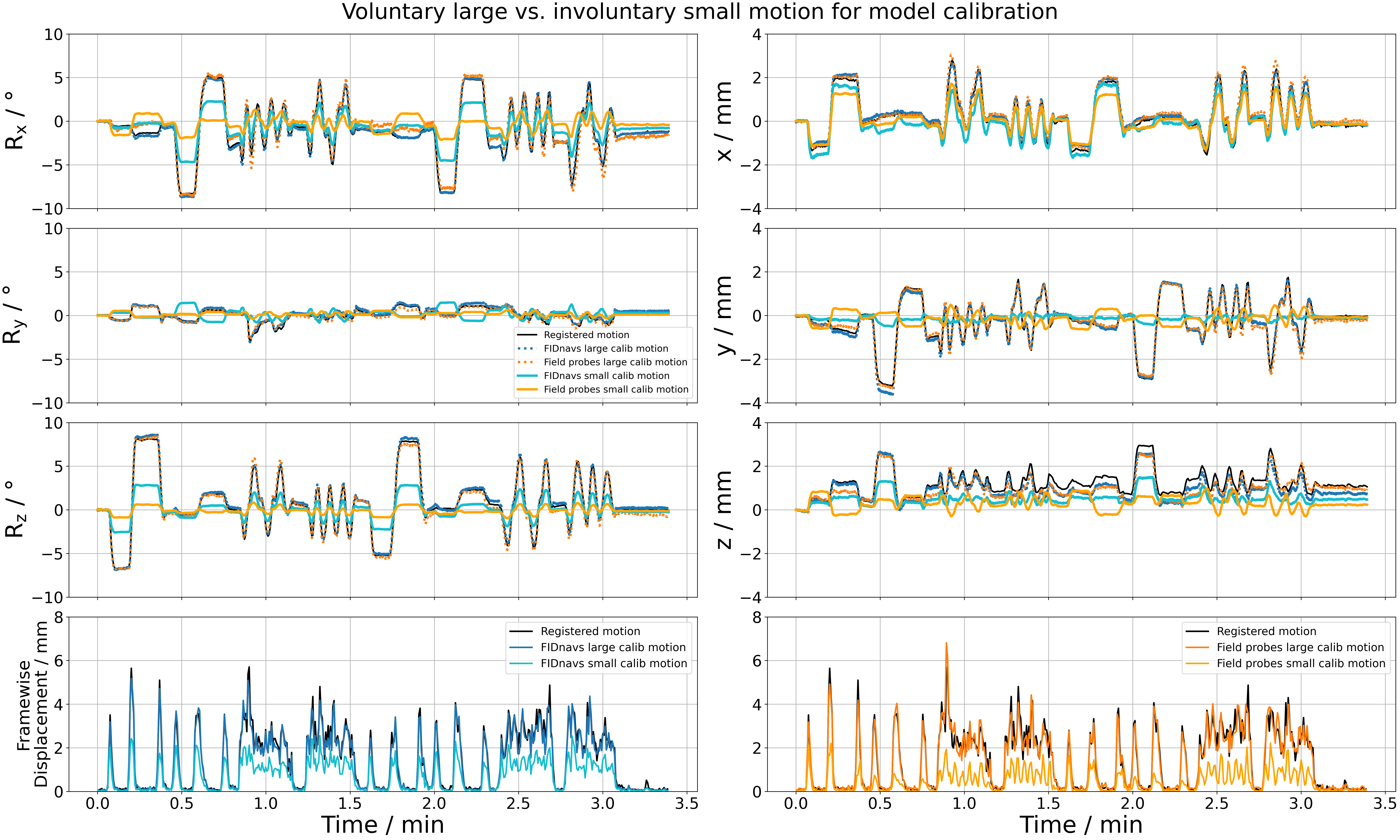

Supplement: Supplementary file 1 — Data S1. Supporting Information. [file MRM-94-105-s001.docx]
